# Photon Counting LIDAR Based on True Random Coding

**DOI:** 10.3390/s20113331

**Published:** 2020-06-11

**Authors:** Yang Yu, Bo Liu, Zhen Chen, Kangjian Hua

**Affiliations:** 1 Institute of Optics and Electronics, Chinese Academy of Sciences, Chengdu 610209, China; yuyang215@mails.ucas.edu.cn (Y.Y.); chenzhen@ioe.ac.cn (Z.H.); huakangjian16@mails.ucas.ac.cn (K.H.); 2 Key Laboratory of Space Optoelectronic Precision Measurement Technology, University of Chinese Academy of Sciences, Beijing 100049, China

**Keywords:** LIDAR, photon counting, true random coding

## Abstract

In this paper, a true random coding photon counting LIDAR is described, in which a Gm-APD (Geiger mode avalanche photodiode) acts as the true random sequence signal generator. The true random coding method not only improves the anti-crosstalk capability of the system, but also greatly reduces the 1-bit missed detection caused by the limited Gm-APD count rate. The experiment verifies the feasibility of the true random sequence used in a photon counting LIDAR ranging system, and a simple and intuitive evaluation model of true random sequence autocorrelation is proposed. Finally, the influence of system parameters (mean echo photon number, mean pulse count density, sequence length, mean noise count) on detection probability is explored. In general, this paper proves that the true random code photon counting LIDAR is an effective target detection method, and provides a new idea for the research of an anti-crosstalk LIDAR system.

## 1. Introduction

The photon counting LIDAR (light detection and ranging) has attracted the attention of researchers and is widely used in the fields of ranging and 3D imaging [1,2,3,4]. Gm-APD (Geiger mode avalanche photodiode) is commonly used as the detector in photon counting LIDAR systems for its weak signal detection capability. However, it has two obvious disadvantages. Firstly, Gm-APD is a two-value output detector that does not respond to the intensity information of the echo signal. This makes Gm-APD highly vulnerable to noise. Meanwhile, it is difficult to extract the target position with a single pulse, so it is necessary to accumulate multi-pulses to establish a statistical histogram. Secondly, when the Gm-APD is triggered, it must be quenched actively or passively, and the quenching process typically lasts for dozens of nanoseconds or even microseconds [5,6]. During quenching, Gm-APD cannot register any arriving photons. This quenching time is called dead time, which causes the nonlinear effects of Gm-APD.

Due to the first disadvantage of Gm-APD, it needs to accumulate enough photons to establish statistical histograms. However, the multi-period cumulative process greatly slows down the measuring speed, and it is not appropriate for scenarios that require quick measurements. In order to solve this problem, Du [7], and Liang [8] demonstrated a high-speed photon-counting laser ranging system with laser pulses of multiple repetition rates to extend the unambiguous distance and achieve fast detection. Yu [9] proposed a method of macro-pulse coding for fast detection of long-distance and high-speed moving targets. Zheng [10] proposed the frequency-multiplexing multi-beam LIDAR which uses 16 independent laser sources with different frequencies and phases to achieve fast detection. 

Photon counting LIDARs based on random coding can also achieve fast detection. This paper will focus on this kind of method. In 1983, the pseudo-random modulation coding method was introduced into the linear detection radar system by Takeuchi for the first time, and it is verified that the pseudo-random coding method can effectively improve the signal-to-noise ratio [11]. The pseudo-random coding method was introduced into the photon counting laser altimeter system by Sun to realize the long-distance target ranging [12]. Hiskett [13], Krichel [14] and Ullrich [15] used a finite non-periodic pulse train or pulse-position modulation technique to resolve range ambiguity. Using the pseudo-random coding photon counting LIDAR ranging technique, Zhang realized the detection of a non-cooperative target at 1.2 km [16]. Yang realized high spatial resolution detection using a high speed pseudo-random modulation photon counting fiber laser ranging system and verified that the ranging accuracy and SNR (Signal Noise Ratio) were improved with the increase in pseudo-random sequences. [17]. In addition, Zhang proved that the random coding method can effectively improve the detection speed compared with the pulse accumulation photon counting LIDAR [18,19]. 

The pseudo-random coding photon counting LIDAR is the combination of the pseudo-random coding method and the photon counting ranging technique. The pseudo-random coding method improves the anti-noise capability of the photon counting LIDAR. Meanwhile, a single-period pseudo-random sequence can extract the target range, which greatly promotes the measurement speed. In addition, the pseudo-random method greatly enlarges the unambiguous distance.

Differing from pseudo-random modulation, we propose a more innovative coding method that can be used for the photon counting LIDAR, called the true random coding method. In the proposed method, a Gm-APD is used as a random signal generator. Our previous work [20] verified the feasibility of the true random coding method through imaging experiments, but there is no in-depth analysis of the advantages and implementation principles. This article will focus on the nature of the true random encoding method. In the following sections, we first elaborated the advantages of the true random coding method, then established the ranging model, then confirmed the feasibility of this method, and finally verified its detection performance by experiments.

## 2. Advantages of the True Random Coding Photon Counting LIDAR

### 2.1. Compared with Pseudo-Random Sequence

Zhang [16] and Yang [17] used the m-sequence as a pseudo-random modulation signal, which requires the signal generator and laser source to have a GHz modulation frequency. High modulation frequency puts forward higher requirements for the signal generator and the laser source. In addition, we can assume that the duty cycle of the m-sequence is approximately 50%. For the m-sequence of bit width 1 ns, it can be considered that the modulation frequency of the transmitted pulse is approximately 500 MHz, which is much higher than the saturated count rate of Gm-APD. Due to the limitation of Gm-APD count rate, the m-sequence has a very high bit error rate. In other words, due to the limited Gm-APD count rate, some bits may not be detected. When extracting the target distance by correlation operation, the missed bits cannot be matched correctly, which will seriously affect the correlation operation results, and thus have a negative impact on the detection performance.

Since the true random sequence is generated by Gm-APD, the pulse frequency of the true random sequence is always lower than its saturation count rate. Therefore, compared with the pseudo-random sequence, the true random sequence effectively avoids the problem of missed detection.

However, some researchers may think that adding a certain number of 0-bits after pseudo-random sequence 1-bits can also solve this problem [21]. We refer to the pseudo-random sequence after filling 0-bits as the modulation pseudo-random sequence. This section will compare the detection performance of the modulated pseudo-random sequence obtained by two different 0-bit compensation methods. The two 0-bit supplement methods are used to insert a fixed number of 0-bits and a random number of 0-bits, respectively. We refer to the modulation pseudo-random sequence supplemented with a fixed number of 0-bits as the first modulation pseudo-random sequence, and the other as the second modulation pseudo-random sequence. 

### 2.2. Compared with the First Modulated Pseudo-Random Sequence

The first modulated pseudo-random sequence only needs to insert a known and fixed number of 0-bits. This modulated pseudo-random sequence can be obtained by simply adjusting the FPGA (Field Programmable Gate Array) and other hardware devices. It does not significantly increase the generating complexity of the modulated pseudo-random sequences.

**Figure 1:**
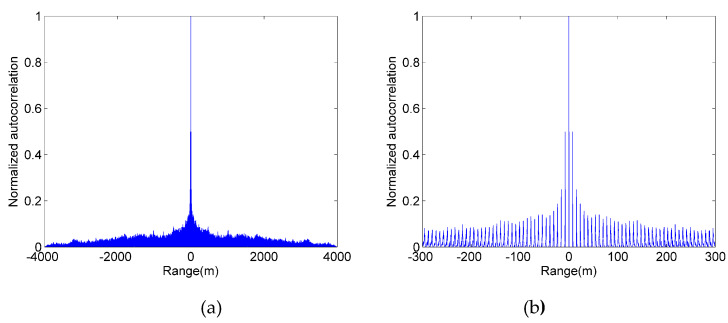
Autocorrelation function of the first modulated pseudo-random sequence. (**a**) global (**b**) local enlarged.

However, the autocorrelation performance of the first modulated pseudo-random sequence is poor. Figure 1a shows the autocorrelation function of the first modulated pseudo-random sequence obtained by inserting 500-bits into the 10-degree m-sequence after each 1-bit. As shown in Figure 1a, this modulated pseudo-random sequence has poor auto-correlation. After zoom-in, as shown in Figure 1b, we can see that the maximum value of the side-lobes is approximately 0.5 in the normalized auto-correlation function. This inherent side-lobe will have a serious negative impact on ranging performance.

The method of replenishing a fixed number of 0-bits is easy to implement in hardware, and it can avoid missed detection due to the limitation of Gm-APD count rate. However, this method reduces the randomness of the original m-sequence due to the insertion of a fixed number of 0-bits. This makes the autocorrelation function have higher side-lobes and is not suitable for ranging systems.

### 2.3. Compared with the Second Modulated Pseudo-Random Sequence

In order to avoid missed detection due to the limitation of Gm-APD count rate and ensure the high autocorrelation of the modulated pseudo-random sequences, random numbers of 0-bits can be added to the m-sequence. In this simulation, 50–100 random unequal numbers of 0-bits are added to the 10-degree m-sequence.

**Figure 2:**
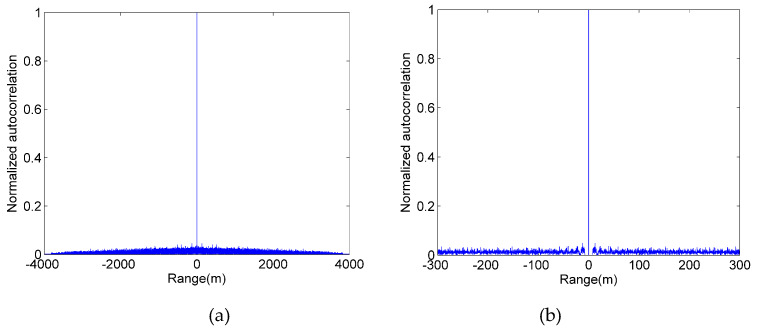
Autocorrelation function of the second modulated pseudo-random sequence. (**a**) global (**b**) local enlarged.

Figure 2a is a global diagram of the autocorrelation function of the second modulated pseudo-random sequence. Figure 2b is the locally enlarged view of Figure 2a. Although the side-lobe value of the autocorrelation function of the second modulated pseudo-random sequence is not 0, it is much smaller than that of the first modulated pseudo-random sequence.

Although the second modulated pseudo-random sequence improves the problem of poor autocorrelation in the first method, the cost of high autocorrelation of the second modulation pseudo-random sequence is that its sequence generation complexity increases significantly. This is because generating the second modulated pseudo-random sequence requires not only the hardware to generate random numbers in real time, but also to adjust the number of 0-bits after each 1-bit in real time. 

It should be pointed out that the idea of the second modulated pseudo-random sequences completely borrows from the true random coding method. Section 3 of this paper will prove that the true random sequence has the same high autocorrelation as the second modulated pseudo-random sequence. However, a Gm-APD can be used to generate a true random sequence of arbitrary pulse count density in real time. Compared with the second modulated pseudo-random sequence, the method of generating a true random sequence does not require a high-bandwidth signal generator and does not require any algorithm processing, making the sequence generation system simpler and more flexible.

### 2.4. Compared with Chaotic Pulse Sequence

In addition to the m-sequence, chaotic signal has attracted the extensive attention of researchers due to its excellent anti-noise and anti-interference capabilities. Du et al. used the chaotic sequence that is only chaotic in the time domain for long-range detection [22]. In order to meet the appropriate chaotic mapping, the chaotic sequence needs to select appropriate parameters. Therefore, the chaotic parameters need to be adjusted appropriately according to different systems. Compared with the true random coding method, the chaos coding method lacks some flexibility.

### 2.5. The Advantage of the True Random Sequence

The true random coding method uses Gm-APD as a random signal generator. The true random electrical pulse sequence generated by Gm-APD drives the laser to directly generate a true random optical pulse sequence for ranging systems. The advantages of this method are mainly reflected in the following aspects.

Firstly, the modulation sequence generated by Gm-APD is a true random sequence. The Gm-APD is triggered by weak continuous light (laser, stray background light) or dark current to generate noise pulses. It is well known that the noise pulse of a single photon detector is completely random. The true random sequence is completely non-repeatable. Thus, it is called a true random sequence, and its confidentiality and anti-crosstalk abilities are remarkably excellent compared with mathematical coding methods (pseudo-random sequence and modulated pseudo-random sequence). Therefore, this true random coding photon-counting LIDAR has obvious advantages in the field of vehicle LIDAR that requires an extremely high anti-crosstalk capability and an extremely high confidential performance. 

Secondly, when the Gm-APD is used as a random signal generator, the dead time characteristic becomes its advantage. The Gm-APD will not generate any pulse during its dead time, which means that the mean pulse count rate of the true random sequence will not exceed the saturation count rate of Gm-APD. In this case, compared with the traditional pseudo-random coding method, the true random coding method can significantly reduce the probability of missed detection of the 1-bit. 

In addition, compared with the modulated pseudo-random sequence and chaotic pulse sequence, only one Gm-APD is needed to generate a true random sequence without any auxiliary equipment or processing algorithms. Therefore, the generation of the true random sequence is simpler and flexible than the modulated pseudo-random sequence and chaotic pulse sequence.

In general, the true random sequence has advantages over the traditional pseudo-random sequence, the modulated pseudo-random sequence and the chaotic pulse sequence in terms of anti-crosstalk ability, ease of implementation and flexibility. Subsequent sections will give a detailed introduction to the true random coding photon counting LIDAR.

## 3. True Random Coding Photon Counting LIDAR Ranging Model

In this part, the system structure and ranging principle are described.

### 3.1. System Structure of the True Random Coding Photon Counting LIDAR System

**Figure 3:**
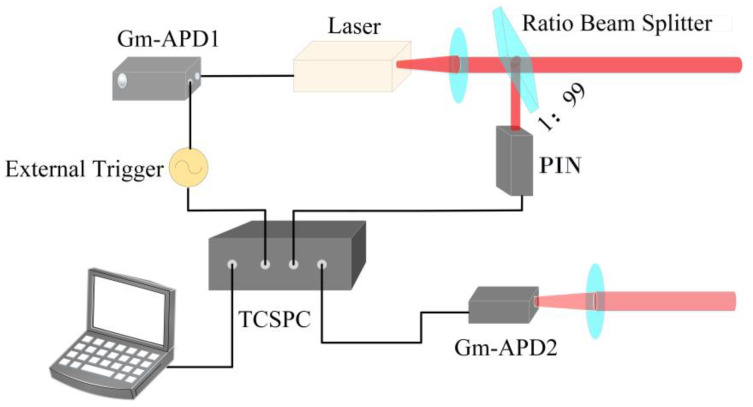
Schematic of the true random coding photon counting LIDAR system.

As shown in Figure 3, two Gm-APDs are used in the system, labeled Gm-APD1 and Gm-APD2, respectively. Gm-APD1 acts as the random signal generator to generate a true random electric pulse sequence. The dead time length of Gm-APD1 should not be less than Gm-APD2 to avoid the limit of Gm-APD2 saturation count rate. Gm-APD2 is used to detect the target’s echo signal.

When activated through the external gate trigger (external trigger), Gm-APD 1 responds to the incident photons and outputs a random electric pulse sequence. The high voltage of the random electric pulse sequence is recorded as the 1-bit. The average number of 1-bits per unit of time is recorded as the mean pulse count density of the true random sequence, and different mean pulse count densities can be obtained by adjusting the optical intensity to meet different experimental requirements. Then, the laser source is modulated by an electrically true random sequence resulting in a laser pulse sequence with equal pulse width and intensity. The true random laser pulses sequence is divided into two parts by a ratio beam splitter: one small part of the energy is detected by a PIN detector, which is used as the transmitted reference signal, recorded as a(n) by TCSPC (time-correlated single photon counting) module; and most of the energy transmits to the target. The echo signal is detected by Gm-APD2 and is recorded as b(n) by the TCSPC module.

### 3.2. The Ranging Principle of the True Random Coding Photon Counting LIDAR System

**Figure 4:**
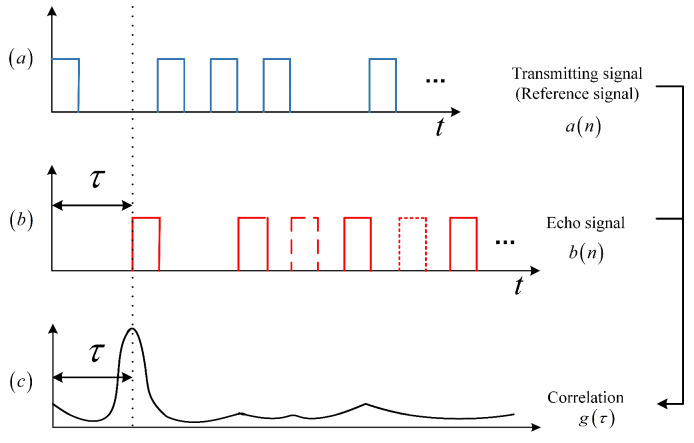
The schematic diagram of the true random coding photon counting LIDAR ranging principle. (**a**) Transmitting signal a(n). (**b**) Echo Signal b(n). (**c**) Correlation function g(τ).

Figure 4 shows that the true random coding photon counting LIDAR extracts the target distance through a correlation operation. Figure 4a–c represent the transmitting sequence, the received sequence (a solid line indicates that the echo signal is detected, a short line indicates that the echo signal is not detected, and a dotted line indicates that noise is detected), and the correlation function, respectively. 

In our system, a(n) represents the true random sequence, which is generated by Gm-APD1 detecting weak continuous stray background light and dark current. a(n) can be written as

(1) $$m_{n1}=\eta_{1}\cdot n_{1}\cdot \Delta t+m_{d1}\cdot \Delta t$$

(2) $$a\left(n\right)=\left\{ \begin{array}{l} \mathrm{sign}\left(1-\sum_{i=1}^{n-1}a\left(i\right)\right)\cdot sign\left(Poisson\left(m_{n1}\right)\right)\;,0<i<n_{d1}\\ \mathrm{sign}\left(1-\sum_{i=n-n_{d1}-1}^{n-1}a\left(i\right)\right)\cdot sign\left(Poisson\left(m_{n1}\right)\right)\;,n_{d1}\leq i\leq N \end{array}\right.,\;n=1,2,3\cdots N$$

$n_{1}$ is the mean photon number received by Gm-APD1. $\eta_{1}$ is the single photon detection efficiency of Gm-APD1. Δt is the bit width. $m_{d1}$ is the mean dark count rate of Gm-APD1. $m_{n1}$ is the mean photoelectron number within the bit width. $Poisson\left(m_{n1}\right)$ generates a random number, which obeys the Poisson distribution with the mean $m_{n1}$. $n_{d1}=\left\lceil t_{d1}/\Delta t\right\rceil$ is the bits number contained in the Gm-APD1’s dead time. $t_{d1}$ is the dead time of Gm-APD1.$N=\left\lfloor T_{a}/\Delta t\right\rfloor$ is the bits number of the true random sequence, $T_{a}$ is the duration when the external trigger signal is high voltage (the length of the true random sequence). We divide the period $T_{a}$ into N bits, and a(n) indicates whether there is pulse count in the nth bit when dead time is considered.

(3) $$m_{s}\left(n\right)=\left\{ \begin{array}{l} 0,a\left(n-\left\lceil \frac{\tau }{\Delta t}\right\rceil \right)=0\\ \eta_{2}\cdot n_{s},\;a\left(n-\left\lceil \frac{\tau }{\Delta t}\right\rceil \right)=1 \end{array}\right.,\;n=1+\left\lceil \frac{\tau }{\Delta t}\right\rceil ,2+\left\lceil \frac{\tau }{\Delta t}\right\rceil ,\cdots N+\left\lceil \frac{\tau }{\Delta t}\right\rceil$$

(4) $$m_{sn}\left(n\right)=\eta_{2}\cdot n_{2}\cdot \Delta t+m_{d2}\cdot \Delta t+m_{s}\left(n\right),\;n=1,2,3\cdots M$$

(5) $$b\left(n\right)=\left\{ \begin{array}{l} \mathrm{sign}\left(1-\sum_{i=1}^{n-1}b\left(i\right)\right)\cdot sign\left(Poisson\left(m_{sn}\left(n\right)\right)\right)\;,\;0<n<n_{d2}\\ \mathrm{sign}\left(1-\sum_{i=n-n_{d2}-1}^{n-1}b\left(i\right)\right)\cdot sign\left(Poisson\left(m_{sn}\left(n\right)\right)\right)\;,n_{d2}\leq n\leq M \end{array}\right.,\;n=1,2,3\cdots M$$

$m_{s}\left(n\right)$ is the mean echo signal photoelectron number within the nth bit. $\eta_{2}$ is the single photon detection efficiency of Gm-APD2. $n_{s}$ is the mean signal echo photon number when the transmitted bit is 1-bit. τ is the time of flight (ToF). $m_{sn}\left(n\right)$ is the mean signal echo and noise photoelectron number within the nth bit. $n_{2}$ is the mean noise photon number of Gm-APD2. $m_{d2}$ is the mean dark count rate of Gm-APD2. $n_{d2}=\left\lceil t_{d2}/\Delta t\right\rceil$ is the bit number contained in the Gm-APD2’s dead time. $t_{d2}$ is the dead time of Gm-APD2. $M=\left\lfloor T_{b}/\Delta t\right\rfloor$ is the number of bits in a period. $T_{b}$ is the period of external trigger signal. $T_{b}$ determines the maximum unambiguous distance of the system.

A correlation function which is similar to Dirac delta function is obtained by performing a correlation operation between a(n) and b(n).

(6) $$g\left(i\right)=\sum_{n=1}^{M}a\left(n\right)b\left(i-n\right)=\left\{ \begin{array}{l} L,i=\pm \left\lceil 2R/\left(c\cdot \Delta t\right)\right\rceil ,\;\pm 2\cdot \left\lceil 2R/\left(c\cdot \Delta t\right)\right\rceil ,\cdot \cdot \cdot \\ \approx 0,i\neq \pm \left\lceil 2R/\left(c\cdot \Delta t\right)\right\rceil ,\;\pm 2\cdot \left\lceil 2R/\left(c\cdot \Delta t\right)\right\rceil ,\cdot \cdot \cdot \end{array}\right.$$

*L* represents the number of 1-bits detected in b(n). *R* is the target distance, and *c* is the light speed. When i=⌈τ/Δt⌉ corresponds to the ToF, the autocorrelation function g(i) has maximum value.

## 4. Autocorrelation Verification of the True Random Sequence 

A true random sequence with high autocorrelation is the basis that can be used for a LIDAR ranging system. In this section, we propose a model to evaluate the autocorrelation performance of the true random sequence based on its basic parameters (mean pulse count density and the length of the true random sequence), and complete the experimental verification.

It can be found by Equation (2) that the true random sequence a(n) can be determined by the density (ρ) of the 1-bit, the length ($T_{a}$) of the true random sequence and the bit width. The bit width mainly affects the system ranging accuracy and has little effect on the autocorrelation. Therefore, we analyze the autocorrelation of the true random sequence in different 1-bit density and sequence lengths.

**Figure 5:**
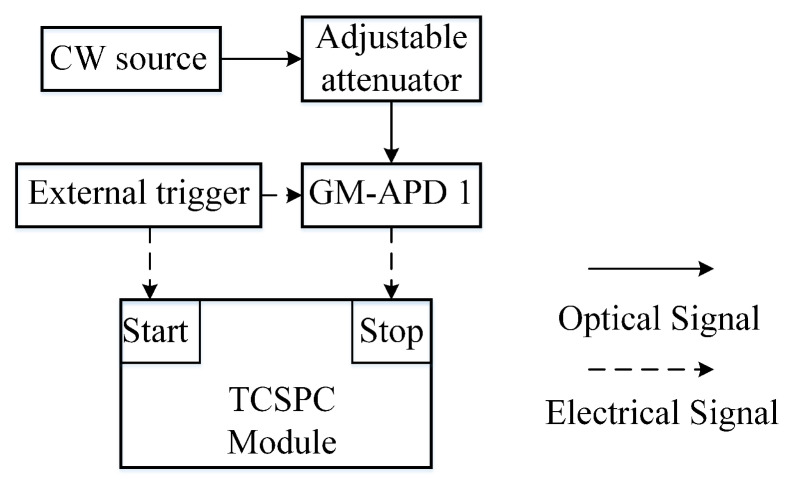
Autocorrelation verification system.

Figure 5 is the autocorrelation verification experimental diagram. The external trigger provides a periodic gate signal to the Gm-APD1, controlling the length of the true random sequence. The density of the true random sequence is adjusted by the intensity of the continuous light.

The autocorrelation function of the true random sequence can be written as [23]

(7) $$\mathbb{R}\left(m\right)=\sum_{n=1}^{N}a\left(n\right)a\left(m-n\right)$$

N is the bit number of the true random sequence a(n). However, it is difficult to intuitively obtain the autocorrelation using the traditional convolutional correlation operation method by the true random sequence’s basic parameters (mean pulse count density, sequence length). In order to solve this problem, a simple and intuitive autocorrelation model is established.

**Figure 6:**
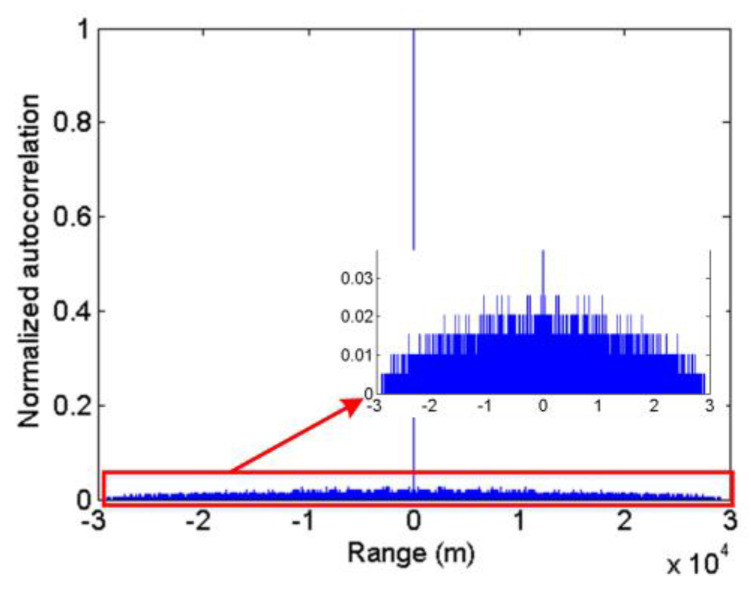
Normalized auto-correlation function of the true random sequence (sequence length 200 μs, mean pulse count density 1 Mcps).

Figure 6 shows a normalized autocorrelation function of a true random sequence. It can be found that the maximum side-lobe value determines the autocorrelation of the true random sequence. A small side-lobe value results in a high autocorrelation. 

Therefore, in the simplified model, we use the ratio of the maximum side-lobe value to the main-lobe as a measure of autocorrelation. The main-lobe (K) is the sum of all 1bits of the true random sequence. The side-lobe (2k) is determined by the maximum number of the same pulse interval in the sequence. Thus, the autocorrelation function can be expressed as

(8) $$\mathbb{R}=\frac{2k}{K}$$

The main-lobe is calculated directly from the sequence length and the mean pulse count density $K=\rho \cdot T_{a}$. The number of the same pulse interval is completely random. In order to obtain its expected value, we perform 10,000 statistics under different sequence lengths and average pulse count densities, and use the average of 10,000 sets of data as experimental results.

**Figure 7:**
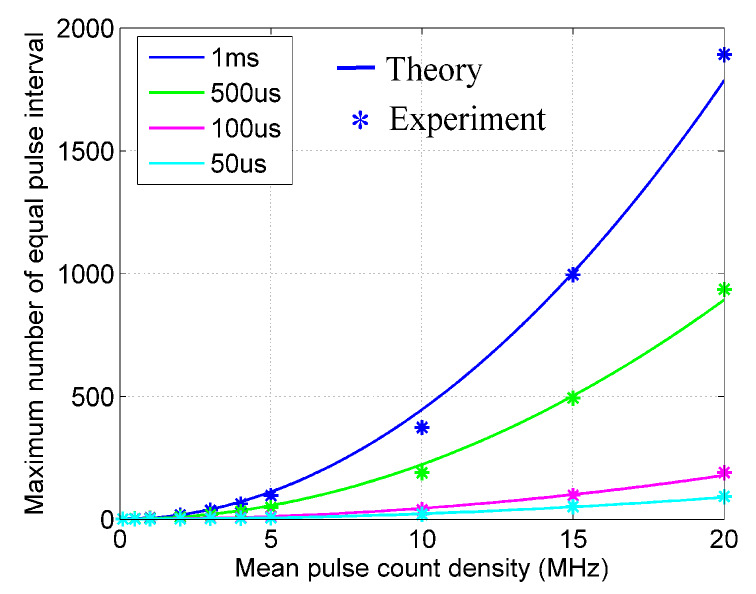
Maximum number of the same pulse interval.

The relationship between the mean pulse count density and the side-lobe (maximum number of the same pulse interval) is found by curve fitting under different sequence lengths. Figure 7 shows the comparison between the theoretical results and the experimental results under the 4 sequence lengths and 10 pulse count densities. The expression of curve fitting is

(9) $$k=\alpha T_{a}\rho^{2}+\beta$$

*k* is the maximum number of the same pulse interval. $T_{a}$ (μs) is the length of the true random sequence. ρ(MHz) is the mean pulse count density. α and β are parameters determined by the Gm-APD.

Substituting Equation (9) into Equation (8), the relationship between the autocorrelation and the sequence parameters (sequence length, mean pulse count density) can be obtained. It can be written as

(10) $$\mathbb{R}=\frac{2k}{K}=\frac{2\left(\alpha T_{a}\rho^{2}+\beta \right)}{T_{a}\cdot \rho }=2\alpha T_{a}+\frac{2\beta }{T_{a}\rho }$$

For the Gm-APD1, α and β are 0.004465 and 1.102, respectively.

**Figure 8:**
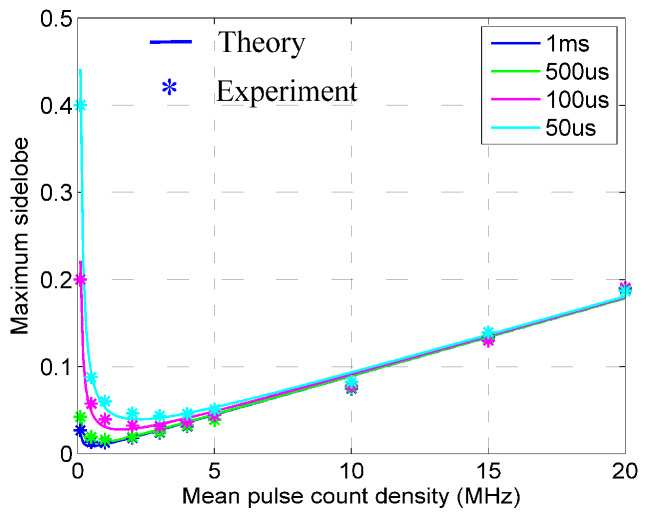
Theoretical and experimental results of the normalized maximum side-lobe.

In Figure 8, side-lobes represent autocorrelation of true random sequences in the simplified autocorrelation function. The smaller the side-lobe, the better the autocorrelation. It can be seen from Figure 8 that the theoretical results and experimental results have a high consistency, indicating that the simplified autocorrelation function model is reasonable. At the same time, it can be found that when the length of the sequence is greater than 50 μs and the mean pulse count density is between 0.5 kHz and 5 MHz, the normalized maximum side-lobe is always less than 0.05, indicating that the true random sequence not only has a higher autocorrelation but also has a higher robustness. Hence, it is feasible to apply the true random sequence for the photon counting LIDAR.

## 5. Experiment Results and Discussion

**Figure 9:**
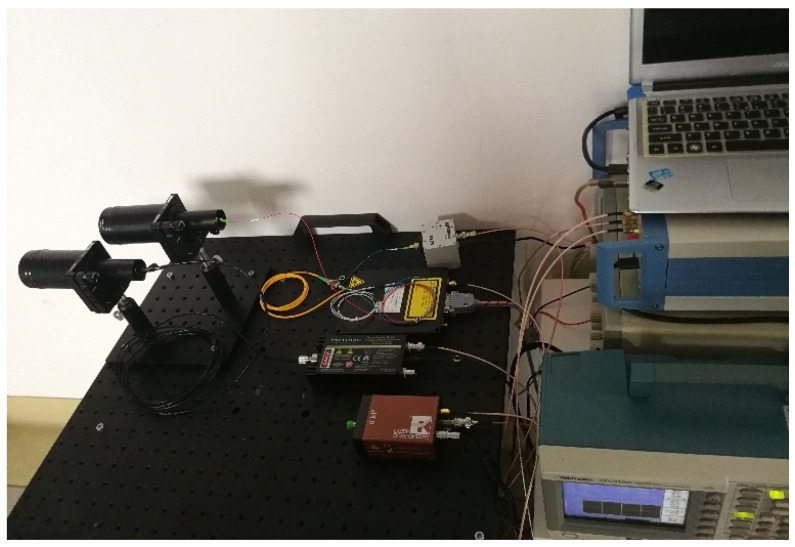
Experimental platform of the true random code photon counting LIDAR.

Section 4 confirms that the true random sequence can be used in the photon counting LIDAR. Then, as shown in Figure 9, we set up an experimental platform to verify the influence of the mean echo photon number, the mean pulse count density and sequence length on the detection probability. The main parameters of the LIDAR system are summarized in Table 1.

**Figure 10:**
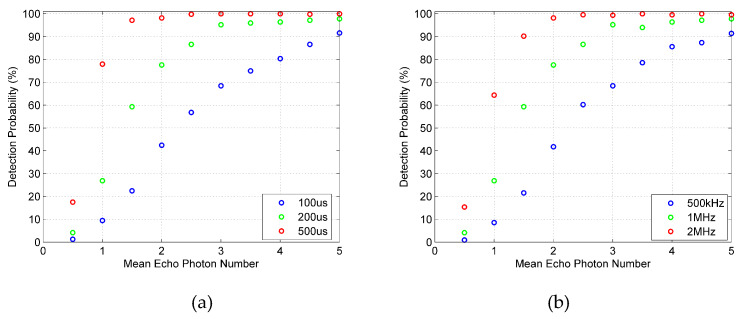
Detection probability of the true random coding photon counting LIDAR. (**a**) Detection probability with different sequence lengths. (**b**) Detection probability with different mean pulse count density.

It can be found in Figure 10 that the detection probability of the true random coding photon counting LIDAR increases with the increase in the mean echo photon number and eventually tends to be saturated. It should be pointed out that the mean echo photon number mentioned in this paper is the mean echo photon number per 1-bit. The single photon detection efficiency of the Gm-APD2 is approximately 2%. When the mean echo photon number of a 1-bit is 1, its mean echo photoelectron is approximately 0.02. When there are only a few echo photons in each 1-bit, the detection probability of a single period true random sequence can reach more than 90%. In general, the proposed true random coded photon counting LIDAR is an effective method for fast detection of weak signals.

Figure 10a shows how the detection probability varies with sequence length (100 μs, 200 μs and 500 μs) under different mean echo photon numbers when the mean pulse count density is 1 MHz and the average noise count is 1 Mcps. It can be seen from Figure 10a that when the detection probability is not saturated, the longer the sequence, the higher the detection probability. However, when the detection probability is close to saturation, the difference of detection probability caused by the change of sequence length is no longer significant. Similar effects of mean pulse count density can be found in Figure 10b. When the detection probability is not saturated, the higher the mean pulse count density, the higher the detection probability. When the detection probability is saturated, the increase in the mean pulse count density will not affect the detection probability.

**Figure 11:**
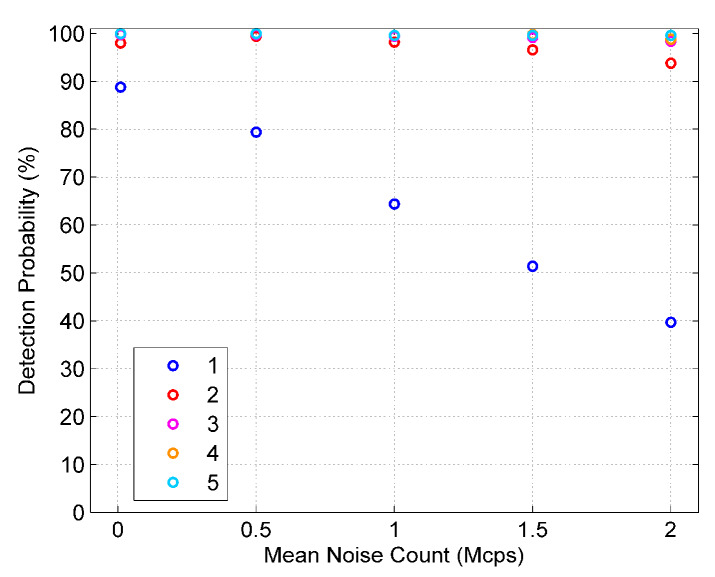
The influence of noise count on detection probability (1, 2, 3, 4, and 5 is the mean echo photon number).

Figure 11 shows the influence of noise count on detection probability under different mean echo photon numbers when the sequence length is 200 μs and the mean pulse count density is 2 MHz. It can be found that when the mean number of echo photons is the same, with the increase in the mean noise count, the detection probability follows a decreasing trend. Meanwhile, if the mean echo photon number is less, the decrease in the detection probability will be more obvious.

The experiment shows that the mean echo photon number, the sequence length, the mean pulse count density and the mean noise count all affect the detection probability. Among them, only the noise count has a negative impact on the detection probability, so we should reduce the noise count of the system as much as possible. At the same time, under a certain echo signal intensity, the experimental results can guide us to select the appropriate sequence length and mean pulse count density to obtain a satisfactory detection probability.

## 6. Conclusions

In this paper, we present a true random coding photon counting LIDAR. The true random sequence has advantages over the traditional pseudo-random sequence, modulated pseudo-random sequence and chaotic pulse sequence in terms of anti-crosstalk ability, ease of implementation and flexibility, especially in the field of anti-crosstalk.

Experiments prove the feasibility of using the true random sequence for photon counting LIDAR ranging systems, and an intuitive performance evaluation model of true random sequences is established. At the same time, the influence of system parameters on detection probability is explored. The experimental results show that the mean echo photon number, the mean pulse count density, the sequence length and the average noise count affect the detection probability. When the detection probability is not saturated, the detection probability is positively correlated with the mean echo photon number, mean pulse count density and sequence length, and negatively correlated with the noise level. Generally, the detection probability can reach more than 90% only when there are several mean echo photons in each 1-bit, which shows that the true random sequence coding photon counting LIDAR can achieve fast and effective weak signal detection. In general, this paper proves that the true random code photon counting LIDAR is an effective target detection method, and provides a new idea for the research of the anti-crosstalk LIDAR system.

## Figures and Tables

**Table 1:** Main Parameters of the LIDAR System.

Parameter	Value
Dead time	45 ns/25 ns
Wavelength	1064 nm
Pulde width	1 ns
Modulation frequency	20 MHz (TTL)
Single photon detection efficiency	1.6%/2%
Time Resolution of TCSPC Module	64 ps
